# *Plasmodium knowlesi* Malaria in Sabah, Malaysia, 2015–2017: Ongoing Increase in Incidence Despite Near-elimination of the Human-only *Plasmodium* Species

**DOI:** 10.1093/cid/ciz237

**Published:** 2019-03-19

**Authors:** Daniel J Cooper, Giri S Rajahram, Timothy William, Jenarun Jelip, Rashidah Mohammad, Joseph Benedict, Danshy A Alaza, Eva Malacova, Tsin W Yeo, Matthew J Grigg, Nicholas M Anstey, Bridget E Barber

**Affiliations:** 1 Menzies School of Health Research and Charles Darwin University, Darwin, Northern Territory, Australia; 2 Infectious Diseases Society Sabah-Menzies School of Health Research Clinical Research Unit, Kota Kinabalu, Sabah; 3 Clinical Research Centre - Queen Elizabeth Hospital, Kota Kinabalu, Sabah, Ministry of Health, Sabah; 4 Sabah Department of Health, Kota Kinabalu, Ministry of Health, Sabah; 5 Gleneagles Kota Kinabalu Hospital, Sabah; 6 Malaysian Ministry of Health, Kuala Lumpur, Malaysia; 7 QIMR Berghofer Institute of Medical Research, Brisbane, Australia; 8 Lee Kong Chian School of Medicine, Nanyang Technological University, Singapore

**Keywords:** knowlesi, malaria, incidence, elimination, epidemiology

## Abstract

**Background:**

Malaysia aims to eliminate malaria by 2020. However, while cases of *Plasmodium falciparum* and *Plasmodium vivax* have decreased substantially, the incidence of zoonotic malaria from *Plasmodium knowlesi* continues to increase, presenting a major challenge to regional malaria control efforts. Here we report incidence of all *Plasmodium* species in Sabah, including zoonotic *P. knowlesi*, during 2015–2017.

**Methods:**

Microscopy-based malaria notification data and polymerase chain reaction (PCR) results were obtained from the Sabah Department of Health and State Public Health Laboratory, respectively, from January 2015 to December 2017. From January 2016 this was complemented by a statewide prospective hospital surveillance study. Databases were matched, and species was determined by PCR, or microscopy if PCR was not available.

**Results:**

A total of 3867 malaria cases were recorded between 2015 and 2017, with PCR performed in 93%. Using PCR results, and microscopy if PCR was unavailable, *P. knowlesi* accounted for 817 (80%), 677 (88%), and 2030 (98%) malaria cases in 2015, 2016, and 2017, respectively. *P. falciparum* accounted for 110 (11%), 45 (6%), and 23 (1%) cases and *P. vivax* accounted for 61 (6%), 17 (2%), and 8 (0.4%) cases, respectively. Of those with *P. knowlesi*, the median age was 35 (interquartile range: 24–47) years, and 85% were male.

**Conclusions:**

Malaysia is approaching elimination of the human-only *Plasmodium* species. However, the ongoing increase in *P. knowlesi* incidence presents a major challenge to malaria control and warrants increased focus on knowlesi-specific prevention activities. Wider molecular surveillance in surrounding countries is required.

## Teaser

Despite near-elimination of *Plasmodium falciparum* and *Plasmodium vivax* malaria in Malaysia, more than 2000 cases of *Plasmodium knowlesi* occurred in the state of Sabah in 2017, accounting for 98% of malaria diagnoses and representing a major challenge to malaria control.

**(See the Editorial Commentary by Karunajeewa and Berman on pages 368–9.)**

Human infections with the zoonotic parasite *Plasmodium knowlesi* occur throughout Southeast Asia in all countries where both the major monkey reservoir (*Macaca fascicularis* and *Macaca nemestrina*) and mosquito vector (*Anopheles leucosphyrus* group) are present [1]. The greatest number of cases have been reported from Malaysia, where *P. knowlesi* has become the most common cause of malaria [2]. *P. knowlesi* is also the predominant species in some areas of western Indonesia [3–5].

Within Malaysia, malaria notification data from the eastern state of Sabah during 1992–2014 demonstrated an increase in the incidence of *P. knowlesi* [6–8]. *P. knowlesi* (and microscopically indistinguishable *Plasmodium malariae*) notifications increased from around 100 per year in the early 2000s, to 703 in 2011 [7], and 1325 in 2014 [8]. In contrast, *Plasmodium falciparum* and *Plasmodium vivax* notifications decreased from a peak of approximately 50 000 in 1994 to 419 in 2014 [7, 8]. Although microscopy is unreliable for distinguishing *P. knowlesi* from other human malaria species [9], a true increase in the incidence of *P. knowlesi* has been suggested by an increase in the proportion of polymerase chain reaction (PCR)–confirmed *P. knowlesi* cases among all malaria notifications in Sabah [6].

Malaysia is aiming to eliminate malaria by 2020, and World Health Organization (WHO) documents report that only 85 malaria cases occurred nationally in 2017 [10]. However, this figure refers exclusively to the human-only *Plasmodium* species, with WHO malaria reports including little information on *P. knowlesi* [10, 11]. *P. knowlesi* is a highly pathogenic parasite with a risk of severe disease in symptomatic adults of 6–9%, at least as high as that of *P. falciparum* malaria in co-endemic settings [12–14], and case fatalities occur [14–18]. In this context, WHO has recognized the need for enhanced regional molecular surveillance to further evaluate the epidemiology and distribution of *P. knowlesi* [19].

We aimed to describe trends in statewide malaria incidence in Sabah using data from the Sabah Department of Health, the State Reference Laboratory, and a prospective malaria surveillance study.

## METHODS

### Setting

**Figure 1.**
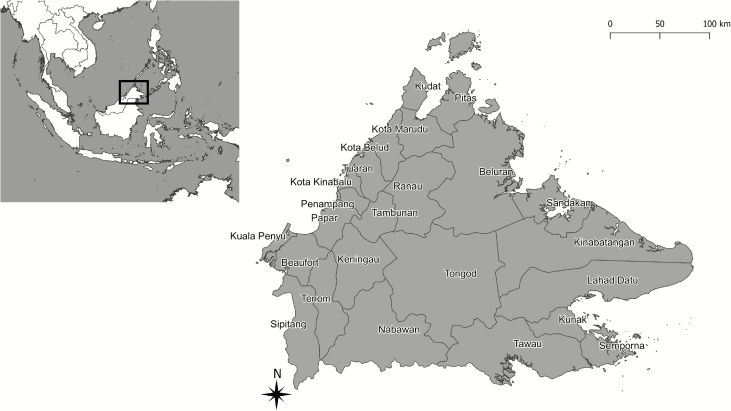
Map of districts of Sabah. The inset shows the Southeast Asia region with the location of Sabah highlighted.

Sabah covers an area of 73 904 km^2^ and is divided into 5 administrative divisions, encompassing 24 districts (Figure 1, Supplementary Table 1). The Crocker range runs along the northeast–southwest axis, with an elevation of 100–4095 m. *A. leucosphyrus* group mosquitoes are present throughout Sabah, as are the natural macaque hosts of *P. knowlesi* [20].

Malaria control strategies include distribution of insecticide-treated bed nets, indoor residual insecticide spraying, and active and passive case detection [21]. Notification of all microscopy-diagnosed malaria cases to the Department of Health remained mandatory throughout the study period in both the public and private sectors, with species reported according to microscopic diagnosis. PCR for all microscopic diagnoses of malaria is performed at the State Public Health Laboratory using methods described previously [22]. The Department of Health mandated free hospital admission and treatment for all patients with malaria throughout the study period.

### Malaria Cases

Microscopy-based malaria notification data and PCR results were obtained from the Sabah Department of Health and State Public Health Laboratory, respectively, from January 2015 to December 2017. From January 2016 this was complemented by a prospective hospital surveillance study, in which standardized data were collected by a nominated hospital staff member for all patients admitted with malaria at every government hospital in Sabah. A unique identifying number was used to match cases across the different data sources. Cross-checking was performed to exclude duplicates and notification entries with a negative *Plasmodium* PCR result, with data then amalgamated in a de-identified database. Final *Plasmodium* species was determined by PCR or by microscopy if PCR was not available (~7% of samples).

This study was approved by the ethics committees of the Malaysian Ministry of Health (NMRR-15-168-24821) and Menzies School of Health Research (HREC-2015–2455).

### Meteorological Data

Monthly rainfall, humidity, and temperature data at the 6 main meteorological sites (Kudat, Kota Kinabalu, Tawau, Sandakan, Ranau, and Keningau) in Sabah were obtained from the Malaysian Meteorological Department from January 2015 to December 2017.

### Statistical Analysis

Data were analyzed using Stata (version 14). Median ages were compared using Wilcoxon rank sum test. Proportions were assessed using chi-square test. Incidence rates were calculated using Malaysian 2010 census district-level population data [23] adjusted by current estimated annual population growth rates [24]. For analysis of age and sex distribution, only PCR-confirmed cases were included. The effects of the average monthly rainfall, humidity, and temperature on the total number of *P. knowlesi* cases per month were evaluated with a lag period of 3 months using a negative binomial model.

## RESULTS

### Malaria Incidence

**Figure 2.**
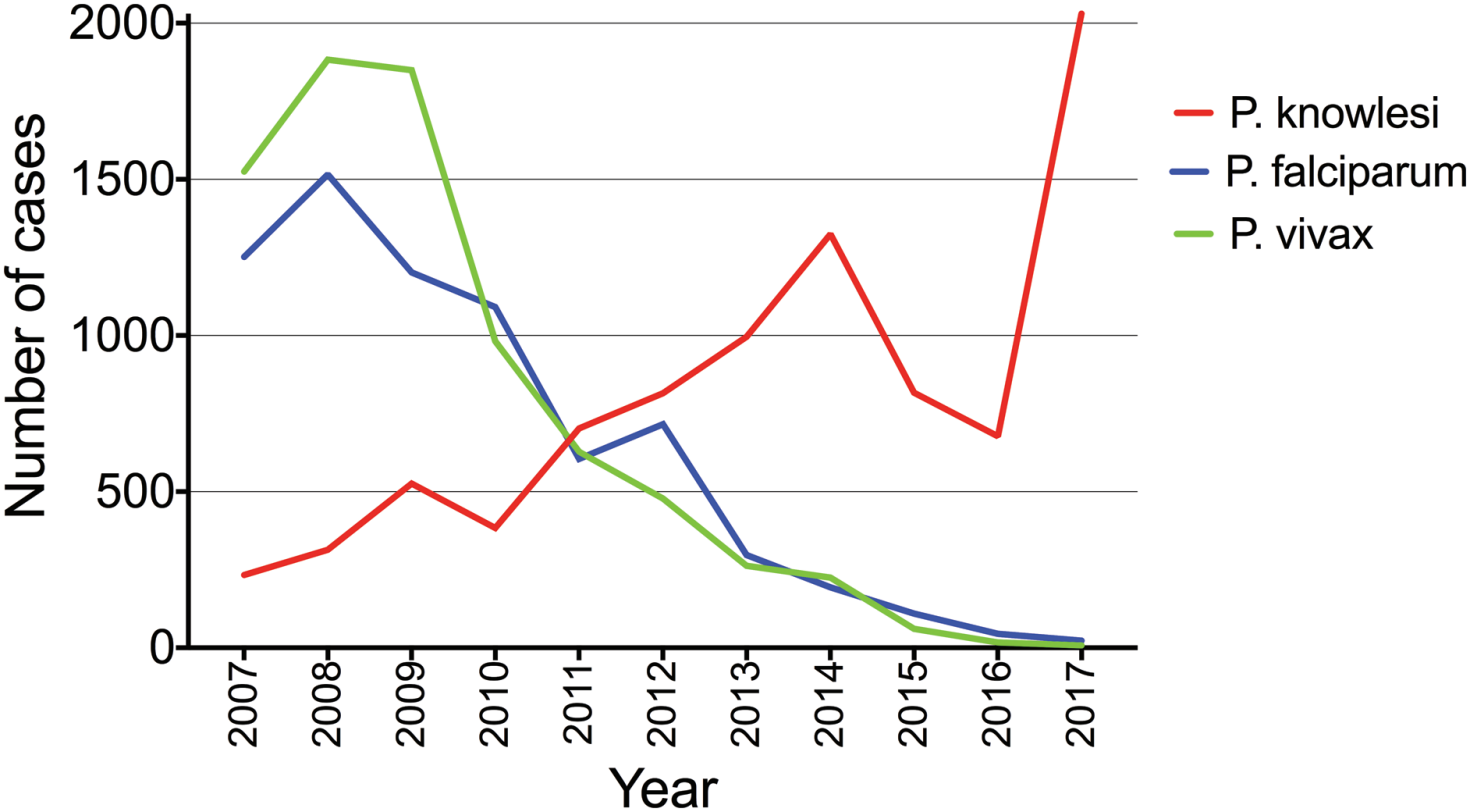
Cases of *Plasmodium knowlesi*, *Plasmodium falciparum*, and *Plasmodium vivax* in Sabah from 2007 to 2017. Data from 2007 to 2014 have been reported previously [6–8].

A total of 3867 malaria cases were recorded during 2015–2017: 1018 in 2015, 771 in 2016, and 2078 in 2017 (Figure 2). Of these, PCR was performed in 99%, 93%, and 91%, respectively. Using PCR results, and microscopy if PCR was not available, *P. knowlesi* accounted for 817 of 1018 (80%), 677 of 771 (88%), and 2030 of 2078 (98%) total malaria cases in 2015, 2016, and 2017, respectively. In contrast, *P. falciparum* accounted for 110 (11%), 45 (6%), and 23 (1%) cases in 2015, 2016, and 2017, respectively, and *P. vivax* for 61 (6%), 17 (2%), and 8 (0.4%) cases.

The *Plasmodium* species distribution was similar when only PCR-confirmed results were used. *P. knowlesi* accounted for 784 of 1001 (78%) PCR-confirmed cases in 2015, 640 of 718 (89%) in 2016, and 1838 of 1880 (98%) in 2017. *P. falciparum* accounted for 96 (10%), 42 (6%), and 23 (1%) PCR-confirmed cases in 2015, 2016, and 2017, respectively; *P. vivax* accounted for 58 (6%), 17 (2%), and 7 (0.4%) cases; and *P. malariae* accounted for 23 (2%), 8 (1%), and 9 (0.5%) cases, respectively. Using PCR, there were 26 (3%) mixed infections in 2015, 9 (1%) in 2016, and none in 2017.

The sensitivity of microscopy for diagnosing *P. knowlesi* against gold-standard PCR was 96% (95% confidence interval [CI], 95–97%) and the specificity was 68% (95% CI, 62–73%) during 2015–2017 (Supplementary Table 1). The positive-predictive value of a microscopy-diagnosed malaria case being *P. knowlesi* by PCR was 97% (95% CI, 97–97%).

### *P. knowlesi* Incidence by District

**Figure 3.**
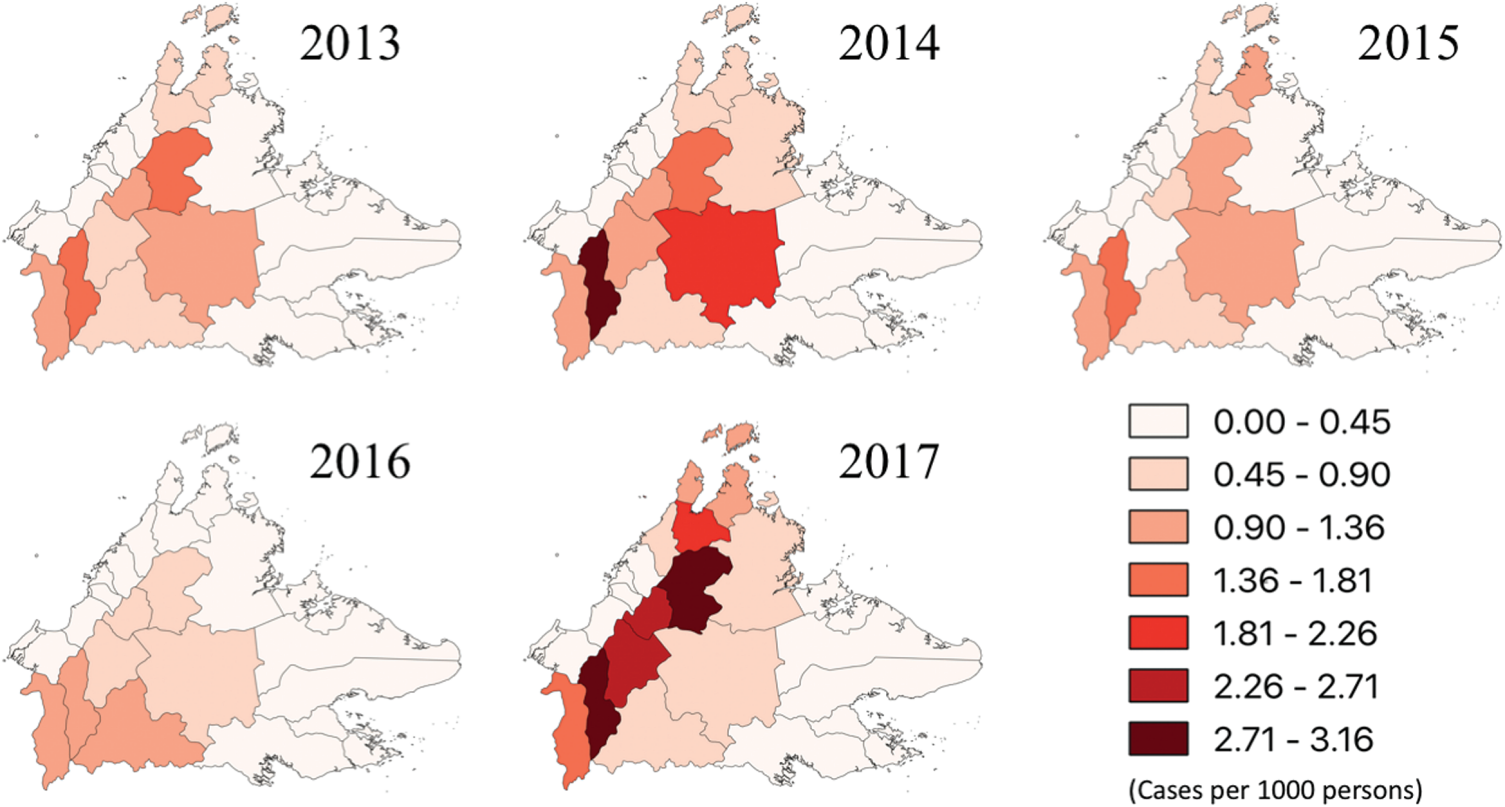
Incidence rate of *Plasmodium knowlesi* by district, 2013–2017. The key indicates *P. knowlesi* cases per 1000 persons.

The majority of districts (18/24; 75%) experienced a decrease in *P. knowlesi* cases from 2015 to 2016; however, the incidence increased in 2017 in all districts except for Kunak (Supplementary Table 2). The highest number of cases occurred in Keningau and Ranau along the Crocker range, with these districts reporting 479 and 358 cases, respectively, in 2017, representing 24% and 18% of the total *P. knowlesi* cases. In Keningau, this represented a more than 5-fold increase from the number of cases observed in 2015. The *P. knowlesi* incidence rate was also highest in these districts (Figure 3). This geographical variation became more pronounced in 2017, with the interior districts reporting a median of 2.1 cases (interquartile range [IQR], 1.4–2.7) per 1000 population compared with 0.1 (IQR, 0.1–0.3) per 1000 population among most East and West Coast districts.

### Age and Sex Distribution

The median age of patients diagnosed with PCR-confirmed knowlesi malaria during 2015–2017 was 35 (IQR, 24–47) years compared with 30 (IQR, 16–41) years for *P. falciparum* (*P* < .0005) and 30 (IQR, 19–39) years for *P. vivax* (*P* = .003). The median age of patients with *P. falciparum* increased from 25 (IQR, 10–37) years in 2015 to 36 (IQR, 25–45) years in 2016–2017 (*P* = .0001; n = 96 and 36, respectively). For *P. vivax*, the median age increased from 28 (IQR, 16–35) years in 2015 to 39 (IQR, 28–55) years in 2016–2017 (*P* = .0007; n = 58 and 24, respectively). The median age of patients with *P. knowlesi* did not change over the study period. There was no statistically significant difference in median age by species for the period 2016–2017.

The majority of *P. knowlesi* cases occurred in adults, with children less than 5 years old accounting for only 25 of 3262 (0.8%) PCR-confirmed infections and children aged 5–13 years accounting for 170 (5%) PCR-confirmed infections. Nonetheless, *P. knowlesi* was the most common cause of malaria in children younger than 14 years, accounting for 195 of 253 (77%) cases. There were 52 PCR-confirmed *P. knowlesi* infections in children younger than 14 years of age in 2015, 36 in 2016, and 107 in 2017. In 2015, there were 30 and 12 children younger than 14 years old with *P. falciparum* and *P. vivax,* respectively, which decreased to 2 and 0, respectively, in 2017.

**Figure 4.**
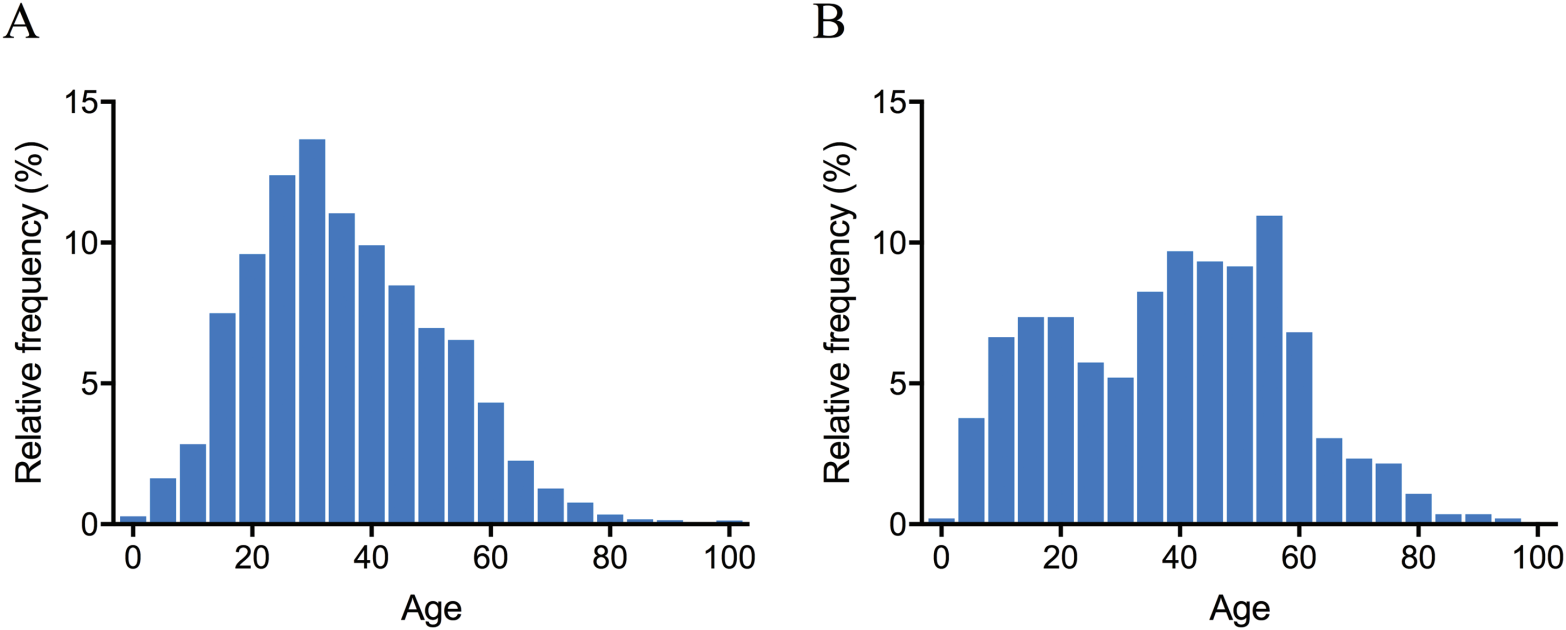
*Plasmodium knowlesi* age distribution, 2015–2017, in males (*A*) and females (*B*).

Males accounted for 2757 of 3263 (85%) PCR-confirmed *P. knowlesi* cases. Females were older, with a median age of 41 (IQR, 23–53) years compared with 34 (IQR, 24–46) years for males (*P* < .0001). Age was normally distributed among males with knowlesi malaria but appeared to follow a bimodal distribution for females (Figure 4).

### Meteorological Data

*There was a small increase in total rainfall at the 6 meteorological stations, from 11 010 mm in 2015 to 18 060 mm in 2017 (* Supplementary Figure 1 *).* In univariate analysis, average monthly rainfall and humidity were both associated with an increased incidence rate ratio of *P. knowlesi* with a lag time of 3 months; however, they were not independent predictors in the final multivariate model (Supplementary Table 3).

### Malaria Deaths and *P. knowlesi* Case Fatality Rate

*Six deaths attributable to malaria were recorded during 2015–2017* [18]*, all of which were confirmed as* P. knowlesi *by PCR. This represented a* P. knowlesi *case fatality rate of* 1.7 (95% CI, .6–3.7) per 1000 cases [18].

## DISCUSSION

This report highlights the ongoing increase in incidence of *P. knowlesi* malaria in Sabah, with 2030 cases occurring in 2017, representing 98% of all malaria cases and the highest annual incidence to date. This has coincided with a decline in falciparum and vivax malaria in Sabah, with only a small number of locally acquired infections occurring in 2017. These continuing trends indicate that the interventions successful at reducing transmission of the human-only *Plasmodium* species do not effectively address the complex factors that are likely contributing to the increase in zoonotic *P. knowlesi* infections.

As with other emerging zoonotic diseases, the increasing incidence of *P. knowlesi* in Sabah is likely driven by changes in human land use, leading to complex changes in transmission of the parasite between humans and the mosquito vectors and the macaque hosts [25]. Sabah has undergone extensive deforestation, with approximately 30% of primary forest area lost between 1973 and 2010 [26], with 21% of Sabah’s total area now used for palm oil plantations [27]. *P. knowlesi* incidence and serological markers of exposure have recently been shown to be associated with higher levels of both forest cover and clearing around households in northern Sabah [28, 29], suggesting that these types of fragmented transition zones are important ecological linkage areas for disease transmission [30]. Modeling has also demonstrated that the relative importance of environmental characteristics associated with *P. knowlesi* transmission, including forest cover and fragmentation indices, aspect, and slope, varies at different spatial scales [29]. Heterogeneity in land-use change between districts is likely to have contributed to the higher incidence of *P. knowlesi* observed in the interior regions of Sabah [30]. Finally, deforestation results in reduced biodiversity, and it is possible that the loss of less-efficient or dead-end hosts has contributed to a higher prevalence of *P. knowlesi* in the highly competent macaque hosts, with consequent spillover of *P. knowlesi* to humans [25].

Deforestation is known to have complex effects on vector bionomics, with malaria transmission potentially increasing as a result of increased sunlight on breeding sites, changing composition of soil, or by changing *Anopheles* species distribution or behavior [31]. In Sabah, *Anopheles balabacensis* is the primary vector of *P. knowlesi* and is found in village, forest, and farming sites [20]. While vectorial capacity remains highest in forested areas [20], *A. balabacensis* has recently been shown to be most abundant in logged areas, with host-seeking behavior more prevalent at ground compared to canopy level [32]. Recent population genetic analyses of *A. balabacensis* across Sabah have demonstrated high genetic variation within subpopulations and a related but currently expanding and growing population overall [33]. Taken together, *A. balabacensis* can be considered a highly competent vector successfully adapting to ecological changes favoring increased zoonotic *P. knowlesi* transmission. However, improvements in predictive *P. knowlesi* risk mapping will also require addressing similar key knowledge gaps regarding the impact of land-use change on macaque distribution and adaptive behavior [25].

Waning immunity to the human-only *Plasmodium* species in Sabah may also be contributing to the increasing incidence of knowlesi malaria. Heterologous immunity was suggested by data from the early malariotherapy studies, with *P. knowlesi* more difficult to induce in patients previously infected with *P. vivax* [34]. This is supported by a study demonstrating that *P. vivax* antibodies can inhibit *P. knowlesi* red blood cell invasion [35]. The possibility that declining cross-protective immunity to *P. vivax* may contribute to rising incidence of knowlesi malaria has important implications for other Southeast Asian countries approaching malaria elimination, highlighting the importance of regional molecular surveillance for *P. knowlesi*. In addition, concurrent monitoring for the presence of other zoonotic *Plasmodium* species should be considered, with human *Plasmodium cynomolgi* infections now reported in West Malaysia, Sarawak in East Malaysia, and Cambodia [36].

In this study, the number of *P. knowlesi* cases decreased between 2015 and 2016, before increasing in 2017. This initial decrease may in part reflect a short-term impact on vector bionomics due to changing rainfall and weather patterns, with *Sabah experiencing unusually low rainfall during this period as part of the worldwide El Niño weather phenomenon*. Consistent with previous reports [6]*, we found an increase in* P. knowlesi *incidence 3 months after higher rainfall. However, this relationship did not remain statistically significant on multivariate analysis, supporting the relative importance of other biotic land-use–related factors driving* P. knowlesi *transmission.* The substantial increase in incidence of *P. knowlesi* in 2017 also raises the possibility of whether human-to-human transmission may now be taking place. To date, phylogenetic analyses of *P. knowlesi* from macaques and humans, in addition to modeling, suggest that transmission remains zoonotic [25, 37]. However, human-to-human transmission has been experimentally demonstrated [38] and, in the context of increasing incidence, cannot be definitively discounted.

As in previous reports, females with knowlesi malaria were older than males [6, 7], and overall, patients infected with *P. knowlesi* were older than those with *P. falciparum* or *P. vivax* [6, 7, 13, 14]. However, in this study there was no difference in the age of those infected with the different *Plasmodium* species when only the period between 2016 and 2017 was considered. This reflects the recent increase in the median age of patients infected with *P. falciparum* or *P. vivax*, as may be expected with waning immunity as these species approach elimination.

Identification of novel interventions targeting specific high-risk population groups or geographical areas will be necessary to control zoonotic *P. knowlesi* transmission. Individual-level acquisition risk is highest in men involved in farming or agricultural work, particularly in palm oil plantations, with other risk factors including sleeping outside, overnight travel, and to a lesser extent, household construction [28, 39]. However, as *A. balabacensis* has adapted to predominantly bite outside in the early evening [20, 39], standard malaria-prevention activities such as insecticide-treated bed nets do not appear to be protective [39]. Promotion of current individual methods of vector protection, including personal insect repellent use for high-risk groups and activities, may be a more appropriate interim strategy.

With over 98% of all malaria cases now caused by *P. knowlesi*, wide dissemination and uptake of case-management protocols for knowlesi malaria are needed in this region. *P. knowlesi* is more likely to cause severe malaria at lower parasitemias than *P. falciparum*, the other major species capable of causing severe malaria, with one district study reporting a 16-fold increase in risk with a parasite count of more than 15 000/µL [12]. Management protocols therefore recommend the prompt use of intravenous artesunate at a lower threshold than that recommended for *P. falciparum* [12, 40]. Longitudinal data from Sabah have previously shown a decrease in case-fatality rate with greater clinical recognition of knowlesi malaria, early referral, and wider use of artesunate [8, 13].

The study had several limitations. Not all patients had PCR performed. However, with nearly all malaria cases now due to *P. knowlesi*, microscopy had a very high pretest probability for diagnosing *P. knowlesi*; thus, the lack of PCR results in a small proportion of patients is likely to have had a minimal impact on our overall results. Second, this study may not have captured all malaria cases, thereby underestimating the total number of knowlesi malaria cases in Sabah during the study period. However, with Department of Health–mandated hospital referral and admission policies for all malaria cases in both public and private sectors, mandatory referral for PCR of all microscopy-positive blood to the State Reference Laboratory, and our surveillance linked to both systems, it is likely that the significant majority of symptomatic malaria cases were identified. Furthermore, with these systems consistent across the study period, it is unlikely to have affected the overall malaria trends demonstrated in the report. The near-elimination of *P. falciparum* and *P. vivax* is supported by contemporaneous cross-sectional community surveillance studies in northeast Sabah [28].

Despite the near elimination of falciparum and vivax malaria in Sabah, over 2000 cases of *P. knowlesi* occurred in 2017, the highest annual incidence to date. *P. knowlesi* thus represents a major challenge to malaria control in Malaysia. With knowlesi malaria now increasingly reported across Southeast Asia, wider molecular surveillance for zoonotic malaria species is needed. Zoonotic *P. knowlesi* will not be eliminated by current measures targeting the transmission of human-only *Plasmodium* species. While efforts must continue to ensure the elimination of the human-only malaria species, current malaria-prevention activities may need to be redesigned, or new approaches developed, to mitigate *P. knowlesi* transmission to humans. In parallel, public health policies need to focus on increasing awareness of knowlesi malaria and ensure that strategies are in place to enable prompt diagnosis and treatment, particularly in high-risk regions.

## Supplementary Data

Supplementary materials are available at *Clinical Infectious Diseases* online. Consisting of data provided by the authors to benefit the reader, the posted materials are not copyedited and are the sole responsibility of the authors, so questions or comments should be addressed to the corresponding author.

ciz237_suppl_Supplementary_Figure_1

ciz237_suppl_Supplementary_Figure_Legend

ciz237_suppl_Supplementary_Tables

## Notes

***Acknowledgments.*** The authors thank the district-hospital nursing staff for data collection and Clarice James for data entry. They also thank the Sabah Department of Health, Ministry of Health, Malaysia, for providing malaria notification data as well as the State Public Health Laboratory (Makmal Kesihatan Awam) for providing polymerase chain reaction data and the Director-General, Ministry of Health, Malaysia, for permission to publish this manuscript.

***Disclaimer.*** The views expressed in this publication are the authors’ alone and are not necessarily the views of the Australian Government.

***Financial support.*** This work was supported by the US National Institutes of Health (grant number R01 AI116472-03); the Australian National Health and Medical Research Council (grant numbers 1037304 and 1045156; and fellowships to N. M. A. [1042072], B. E. B. [1088738], and M. J. G. [1138860]); “Improving Health Outcomes in the Tropical North: A Multidisciplinary Collaboration ‘Hot North’” (grant number 1131932); and the Australian Centre of Research Excellence in Malaria Elimination. D. J. C. is supported by Prestigious International Research Tuition Scholarship and University Postgraduate Research Scholarship from Charles Darwin University. T. W. Y. is funded by a Singapore National Medical Research Council Clinician Scientist Award (CSA INV 15nov007). The study was also supported by the Tropical Disease Research Regional Collaboration Initiative , which is funded by the Australian Government Department of Foreign Affairs and implemented by the Menzies School of Health Research.

***Potential conflicts of interest.*** The authors have no potential conflicts of interest to disclose. All authors have submitted the ICMJE Form for Disclosure of Potential Conflicts of Interest. Conflicts that the editors consider relevant to the content of the manuscript have been disclosed.
