# Supplementary figures and images for: Plasmodium knowlesi Malaria in Sabah, Malaysia, 2015–2017: Ongoing Increase in Incidence Despite Near-elimination of the Human-only Plasmodium Species
Source: Clin Infect Dis. 2019 Mar 19;70(3):361–7. doi: 10.1093/cid/ciz237 (PMC7768742; doi:10.1093/cid/ciz237)

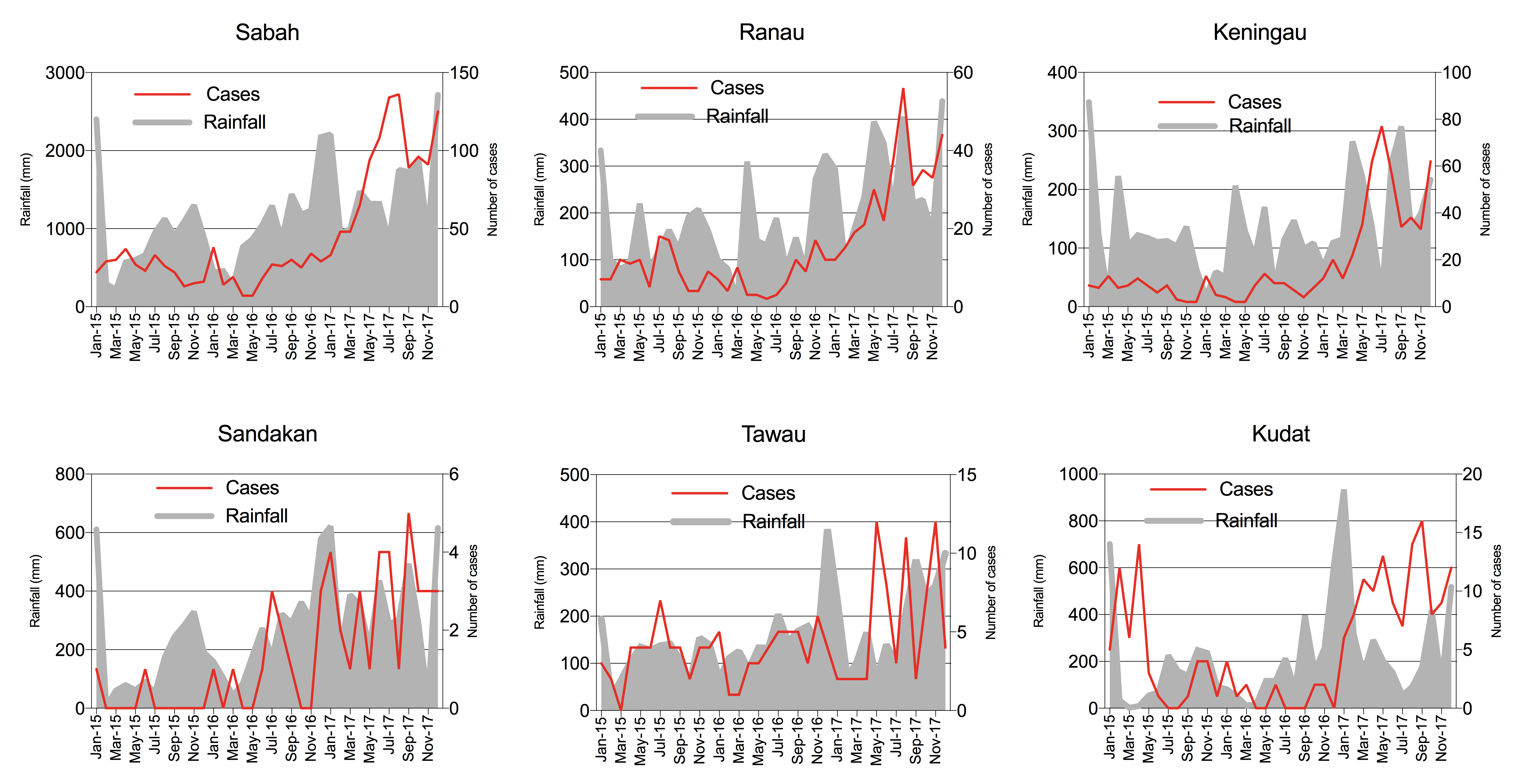

Supplement: ciz237_suppl_Supplementary_Figure_1 [file ciz237_suppl_supplementary_figure_1.png]
