# Supplementary material for: Plasmodium knowlesi Malaria in Sabah, Malaysia, 2015–2017: Ongoing Increase in Incidence Despite Near-elimination of the Human-only Plasmodium Species
Source: Clin Infect Dis. 2019 Mar 19;70(3):361–7. doi: 10.1093/cid/ciz237 (PMC7768742; doi:10.1093/cid/ciz237)
Supplement: ciz237_suppl_Supplementary_Figure_Legend [file ciz237_suppl_supplementary_figure_legend.docx]

Supplementary Figure 1: *P. knowlesi* notifications and monthly rainfall in Sabah (A) and at 5 meteorological stations in Ranau (B), Keningau (C), Sandakan (D), Tawau (E) and Kudat (F) districts.
