# Supplementary material for: Plasmodium knowlesi Malaria in Sabah, Malaysia, 2015–2017: Ongoing Increase in Incidence Despite Near-elimination of the Human-only Plasmodium Species
Source: Clin Infect Dis. 2019 Mar 19;70(3):361–7. doi: 10.1093/cid/ciz237 (PMC7768742; doi:10.1093/cid/ciz237)
Supplement: ciz237_suppl_Supplementary_Tables [file ciz237_suppl_supplementary_tables.docx]

**Supplementary Table 1: PCR results compared with microscopy.**

| **Microscopy** |  |  | **PCR** |  |  |  |  |  |  |  |
| --- | --- | --- | --- | --- | --- | --- | --- | --- | --- | --- |
|  | **Pk** | **Pf** | **Pv** | **Pm** | **Pk/Pf** | **Pk/Pv** | **Pf/Pv** | **Po** | **P. genus** | **Total** |
| **Pk/Pm** | 3164 | 41 | 21 | 38 | 9 | 2 | 2 | 3 | 15 | 3295 |
| **Pf** | 51 | 116 | 2 | 2 | 1 | 0 | 2 | 0 | 1 | 175 |
| **Pv** | 27 | 2 | 57 | 0 | 0 | 1 | 0 | 0 | 0 | 87 |
| **Mixed** | 20 | 2 | 2 | 0 | 13 | 3 | 2 | 0 | 0 | 42 |
|  |  |  |  |  |  |  |  |  |  |  |
| **Total** | 3262 | 161 | 82 | 40 | 23 | 6 | 6 | 3 | 16 | 3599 |
|  |  |  |  |  |  |  |  |  |  |  |

Abbreviations: Pf = *Plasmodium falciparum*, Pv = *Plasmodium vivax*, Pk = *Plasmodium knowlesi*, Pm = *Plasmodium malariae*, Po = *Plasmodium ovale*

**Supplementary Table 2: Number of cases of *P. knowlesi* by district.**

|  |  |  | Cases |  |
| --- | --- | --- | --- | --- |
| Division | District | 2015 | 2016 | 2017 |
| **Interior** |  |  |  |  |
|  | Beaufort | 8 | 6 | 13 |
|  | Keningau | 86 | 107 | 479 |
|  | Kualu Penyu | 0 | 0 | 0 |
|  | Nabawan | 37 | 62 | 53 |
|  | Sipitang | 47 | 56 | 65 |
|  | Tambunan | 33 | 25 | 97 |
|  | Tenom | 98 | 79 | 209 |
|  |  | **309** | **335 (+8)** | **916 (+173)** |
| **Kudat** |  |  |  |  |
|  | Kota Marudu | 50 | 32 | 153 |
|  | Kudat | 49 | 13 | 123 |
|  | Pitas | 46 | 19 | 48 |
|  |  | **145** | **64 (-55)** | **324 (+406)** |
| **Sandakan** |  |  |  |  |
|  | Beluran | 46 | 14 | 76 |
|  | Kinabatangan | 19 | 14 | 27 |
|  | Sandakan | 2 | 15 | 35 |
|  | Tongod | 38 | 25 | 25 |
|  |  | **105** | **68 (-35)** | **163 (+140)** |
| **Tawau** |  |  |  |  |
|  | Kunak | 10 | 10 | 5 |
|  | Lahad Datu | 38 | 33 | 78 |
|  | Semporna | 4 | 1 | 3 |
|  | Tawau | 41 | 40 | 57 |
|  |  | **93** | **84 (-10)** | **143 (+70)** |
| **West Coast** |  |  |  |  |
|  | Kota Belud | 14 | 7 | 62 |
|  | Kota Kinabalu | 1 | 10 | 15 |
|  | Papar | 18 | 7 | 15 |
|  | Penampang | 7 | 8 | 16 |
|  | Ranau | 114 | 89 | 358 |
|  | Tuaran | 11 | 5 | 18 |
|  |  | **165** | **126 (+24)** | **484 (+284)** |
|  |  |  |  |  |
|  | **TOTAL** | **817** | **677** | **2030** |

Numbers in parentheses represent percentage change from previous year.

**Supplementary Table 3: Association between average monthly rainfall, humidity and temperature, and incident rate ratio of *P. knowlesi* malaria, 2015 – 2017.**

|  | Univariate analysis | | Multivariate analysis* | |
| --- | --- | --- | --- | --- |
|  | IRR (95% CI) | p-value | IRR (95% CI) | p-value |
| Time (in months) | 1.059 (1.042-1.076) | <0.001 | 1.055 (1.035-1.076) | <0.001 |
| Average rainfall (mm) | 1.004 (1.002-1.007) | 0.004 | 1.001 (0.998-1.004) | 0.732 |
| Average humidity (%) | 1.203 (1.102-1.314) | <0.001 | 1.004 (0.873-1.155) | 0.955 |
| Average temp (°C) | 0.643 (0.408-1.014) | 0.057 | 0.790 (0.562-1.110) | 0.174 |

*Adjusted for all other variables

Abbreviations: IRR = Incidence rate ratio, CI = confidence intervals

In the univariate analysis, the incident rate of total number of malaria cases increased by 6% for every additional month, increased by 0.4% for every unit increase in average rainfall, increased by 20% for every unit increase in average humidity, and decreased by 36% for every unit of increase in average temperature. After mutually adjusting for all variables, only time (months) remained statistically significant.
